# Owning, Renting and Environmental Proactivity: The Role of Housing Tenure in Hypothetical Housing Decisions

**DOI:** 10.1177/00469580251370562

**Published:** 2025-09-15

**Authors:** Manuela Schulz, Christiane Gross, Andrea Teti

**Affiliations:** 1 University of Vechta, Germany; 2 University of Würzburg, Germany

**Keywords:** ownership, residential decision-making, environmental proactivity, social inequality, factorial survey

## Abstract

Past research indicates that for older individuals, transitioning to a home environment better suited to their needs reduces physical, psychological, and social risks, and may even impact the rate of institutionalization. Tenants, compared to homeowners, are subject to different conditions that influence their decisions to relocate, which can either encourage or inhibit them in their pursuit of environmental proactivity. This study investigates whether tenants make relocation decisions based on different factors than do owners. For this purpose, hypothetical relocation decisions are made under the influence of certain ownership constellations. The dataset consists of 264 participants. They were asked about home ownership and then presented with housing vignettes (factorial survey) to indicate how likely they would be to move to a new apartment. The data were analyzed using group comparisons and zero-inflated models. Tenants favor new apartments if their current dwelling is larger than the new one, and if they haven’t lived in their current home for a long time. In contrast, owners prefer the new apartment to have a central location. Both groups consider rent, proximity to kin, and a senior-friendly bathroom as important for the new apartment, with rent being more important to tenants than to those who are currently owners. In both groups, we identified predictors that can be interpreted as barriers to environmental proactivity. The results add to the large body of literature on social inequality in old age.

**Highlights**

● Explores how housing tenure shapes older adults’ hypothetical relocation choices.

● Applies factorial survey design to examine downsizing in later life.

● Identifies barriers to environmental proactivity among tenants and owners.

● Situates findings within German housing policy and international debates.

## Introduction

### Environmental Proactivity and Residential Decision-Making

Housing plays a fundamental role in shaping well-being across the life course, particularly in later life.^1,2^ Recent research has emphasized the importance of person–environment fit, that is, the alignment between individual needs and environmental conditions, as a key determinant of quality of life in older age.^3-5^ Lawton’s concept of environmental proactivity emphasizes the active role individuals can play in shaping their environment to maintain or enhance their well-being. Rather than passively adapting to constraints, older adults can “*seek, choose, or create an environment in order to satisfy [their] needs and preferences*.”^4^

The process of residential decision-making was also discussed in the context of environmental proactivity.^4^ Residential decision-making is understood as a proactive process in which older adults reflect on their current housing situation and make deliberate choices about whether and how to relocate. In this sense, residential choices are not merely responses to decline but can reflect agency and future orientation.^6-8^

### Narrowing the Field of Research

In this study, we deal with voluntary home-to-home moves to rented apartments (not houses) that are smaller than the one currently occupied (Downsizing, see Refs.^9,10^). Moving to a care facility is fundamentally different from moving from one home to another, and this topic has been addressed elsewhere (see, eg, Winke^11^ or Marshall et al^12^). Angelini and Laferrère describe in detail the differences between relocations with and without a change of tenure in both directions.^10^ We decided to focus on people moving into a rented dwelling (from a status of tenant or owner-occupier) for the sake of clarity, as the acquisition of a new dwelling involves additional considerations.

### Decision to Relocate as a Preventive Measure

The most common outcome of residential decision-making processes is that people decide to stay in their already familiar environment, often despite there no longer being a person-environment fit.^13,14^ However, research in recent years has shown that moving in old age to a home more suited to one’s abilities reduces the risk of accidents and injuries, results in less social isolation, depression, and anxiety, and reduces the risk of institutionalization.^13^ The initially negative association between moving and health has been refuted in the past. As a result, the paradigm of “aging in place”—once synonymous with remaining in the same home at all costs—has begun to shift.^2,13,15^ Importantly, aging in place is no longer solely defined by the physical continuity of dwelling, but also by the preservation of autonomy and a sense of embeddedness within one’s community (eg, during COVID-19^16^). From this perspective, relocation can, in some cases, support rather than threaten older adults’ independence, particularly when it enables access to age-appropriate housing, social networks, or essential services.^15^ The German housing market is currently marked by a sustained excess in demand, particularly in urban areas, driving up rents, and property prices.^17^ While housing policy aims to ensure affordability and access across all social groups, older adults occupy a substantial share of the existing housing stock.^18^ However, much of this housing is poorly suited to the needs of aging residents—it is often too large, physically inaccessible, or lacking age-appropriate amenities—highlighting a critical mismatch between the living environment and the functional requirements of later life.^19^ Although the share of informal caregiving at home is lower in Germany than in other European countries, German policy prioritizes support for home-based and informal care, viewing institutionalization primarily as a last resort.^20^ In Germany, policy initiatives increasingly promote age-appropriate housing and services within familiar neighborhoods to support autonomy and social inclusion, including relocation within one’s community when needed (eg, “seniorengerechte Quartierskonzepte”^21^ or “Wohnen.Pflege.Nachbarschaft”^22^). These developments are closely related to core concerns in health services research, such as equity in access to age-appropriate housing and the organization of (informal) care structures that support autonomy in later life.^23^ From a health policy perspective, relocation decisions should be viewed not only through the lens of individual preference but also as indicators of systemic capacity to enable independent aging in safe, affordable, and supportive environments.

### Housing Tenure as an Influencing Factor

Housing decisions in old age depend on several factors, such as current housing conditions and the expected benefits of moving.^6,8^ International comparisons suggest that residential decision-making processes are influenced by factors related to socio-economic status and economic conditions^10,24^ and are shaped by structural mechanisms of inequality, including but not limited to race and ethnicity, gender, and financial situation.^25^ Therefore, housing tenure appears to be the obvious variable to examine when researching residential decision-making processes. Owning a home comes with considerable benefits. Studies have shown that home ownership increases life satisfaction.^26^ Owners are also physically healthier,^27^ report better mental health,^28^ and live longer than tenants.^29^ Some of these health inequalities may be due to financial aspects. Tenants have a 3 times higher risk of poverty and a particularly high relative housing cost burden.^30-33^ In addition, the relative housing cost burden for tenants continues to increase over their life-course, which is not the case for owners.^32^ In addition to these disadvantages in terms of finances, health, and even mortality, tenants also appear to be worse off in a direct comparison of housing conditions. Neighborhoods with a high proportion of rented housing are affected more quickly and to a greater degree by demographic or social changes, which might lead to a feeling of social exclusion and social withdrawal.^34^ Furthermore, tenants are only able to adapt a dwelling to their own needs to a limited extent, as this can only be done by or in consultation with the landlord. A study by Oswald et al showed that tenants have greater external housing control beliefs, that is, compared with owners, they believe that their living environment is more controlled by external factors.^35^

These factors are likely to influence tenants’ housing decisions in a way that runs counter to the idea of environmental proactivity, and they may prevent older people from utilizing environmental proactivity as a strategy for successful aging (see also Ref.^24^). However, there may also be factors that inhibit environmental proactivity among owners, such as a lack of flexibility and a greater financial commitment to one’s home.^10^

### Aims of This Study

A systematic literature review by Roy et al identified 660 titles that addressed the factors influencing the housing decisions of frail older adults. However, of these 660 titles, only 4 dealt with the different tenure types, of which only 2 are currently less than 20 years old.^10,36-39^

In the late 1990s and early 2000s, VanderHart examined housing tenure choice from an economic perspective and found that home equity, income, and age have a significant impact on the tenure decisions of older adults.^38,39^ Granbom et al investigated the predictors of moving into ordinary or special housing over a 4-year period at a very old age (80-89 years). They found that owner-occupiers were more likely to move into ordinary housing compared to tenants. Tenure status had no predictive value for moving into special housing.^37^ Angelini and Laferrère investigated predictors of relocation in groups, focusing on tenure status and changes in tenure during relocation. Their findings indicate that individuals transitioning from home ownership to renting base their decisions on income, wealth, and household size. They were more likely to move if they were divorced and did not live in a rural area. In contrast, those moving from one rental to another based their decisions on the duration of residence, wealth, and changes in their economic situation.^10^

In this study, we aim again to describe how owners and tenants differ in their housing choices. To our knowledge, no study has employed a factorial survey design to investigate predictors of relocation based on tenure status in old age. This methodological approach enables participants to evaluate multiple housing options simultaneously, allowing for a comprehensive analysis that incorporates both current and future housing characteristics as potential determinants of relocation decisions.

We defined 3 groups of potentially influential predictors (predictors describing the current housing situation, current and expected financial burdens, and characteristics of the future/hypothetical apartment). We do not expect certain associations to necessarily be reversed between groups (eg, we do not expect owner-occupiers to want a home full of barriers while tenants do not); however, we do expect some associations to be stronger in one of the 2 groups. Note again that in this study, we are dealing with hypothetical relocations to smaller apartments (since downsizing is very common in old age), which are rented (to reduce complexity). The focus on apartments rather than houses was a deliberate design choice, both to align with typical downsizing patterns in Germany, where older adults often move from larger houses to smaller apartments,^9^ and to reduce complexity in our vignette design. In a second step, we aim to discuss which of these differences can be viewed as a disadvantage in the context of environmental proactivity. Understanding how housing systems can foster or hinder adaptive strategies, such as environmental proactivity, helps inform policies aimed at reducing health disparities and improving long-term care outcomes. Beyond contributing to the empirical understanding of housing preferences in later life, this study also aims to provide conceptual insights relevant beyond the German context, particularly regarding how tenure status influences opportunities for proactive environmental adaptation in aging populations.

### Hypotheses

**Figure 1.**
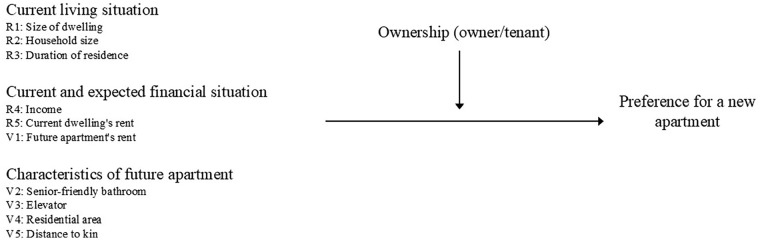
Hypotheses.

We formulate hypotheses at the respondent level (R1-R5) and the vignette level (V1-V5; Figure 1).

#### Current Housing Situation

Although parting with identity-forming possessions and familiar environments can be emotionally challenging,^13^ the desire for a smaller and more manageable dwelling is among the top 3 reasons older adults report for past or planned relocations,^9^ most likely due to mobility limitations and difficulties maintaining ample space. For individuals in the early stages of old age—often referred to as the *young-old* (approximately aged 60-79)—a larger home can continue to be a source of satisfaction, autonomy, and symbolic continuity. In contrast, for the *old-old* (typically aged 80 and above), increasing physical or cognitive limitations may turn spacious dwellings into sources of stress or risk. Prior research has linked larger homes to lower life satisfaction among the old-old.^1^ On average, the chosen dwelling becomes 0.3 to 1.4 rooms smaller with age, and is especially likely to reduce in size if the currently-occupied dwelling has a large number of rooms.^10^ As owner-occupiers often live in larger dwellings,^2,31^ we expect the association to be particularly high for them.

*R1: The strength of preference for a new, smaller home is greater when the current dwelling is larger (especially for those who are currently owner-occupiers).*

Widowhood, and also divorce, have promoting effects on residential mobility because resources and the environment are adapted to the new situation when less living space is needed due to a smaller household size.^9,10,40^ We also expect this effect to be more substantial for owners, as there may be explicit or implicit agreements to pass on the dwelling to the next generation, who are more likely to have greater spatial requirements.^41,42^

*R2: The strength of preference for a new, smaller home is greater when the household size is low (especially for those who are currently owner-occupiers).*

People feel a strong attachment to their living environment. This is associated with identity-forming features of the home and subjectively valued memories. Previous research has shown that the longer a person lives in a home, the greater the perceived attachment to it and the subjectively valued quality of living.^1,2,43,44^ This correlation suggests that individuals who have lived in their homes for a long time are more likely to refuse to move. We could not find any indication that the duration of residence might have different effects on the housing decision process for tenants and owners.

*R3: The strength of preference for a new, smaller home is greater when the duration of residence in the current home is shorter.*

#### Current and Expected Financial Burdens

As not only is the income of owners often higher than that of tenants, but also because the housing costs are higher for tenants than for owners, and represent a higher share of income,^30^ we expect the housing costs of the current dwelling to play a significant role, especially for tenants.

*R4& R5: Tenants’ strength of preference for a new, smaller home is greater when the income is low and the rent in the current dwelling is high.*

Even though it is one of the 3 most frequently cited reasons for moving in old age, moving to a smaller home after many years is not necessarily associated with a reduced rent, due to increases in the cost of housing and the fact that older people have often lived in their previous homes for a long time,^9,10^ which is reflected in a discrepancy between planned and actual moves among older tenants.^9^ Thus, in addition to the push factor of high current rent, the pull factor of lower new rent is also needed for a tenant to decide to move. The rent of the new dwelling presumably also plays a role for owners, but to a lesser extent, due to the higher income and the presumably freed-up capital.

*V1: The strength of preference for a new, smaller home is greater when the new rent is lower (especially for tenants).*

#### Characteristics of Future Apartments

Keeping the home functional for everyday life and maintaining one’s independence is one of the most common reasons for redesigning the home environment^6,45^ or moving to a smaller home.^8,9^ We therefore expect all participants to agree more readily to an apartment that meets basic needs (eg, an accessible bathroom, a lift). According to the retirement migration model, people can restore the person-environment fit without moving by making housing adjustments.^8^ Tenants are inherently constrained by their housing situation in making environmental changes. For example, significant structural alterations (such as widening the doorway) are sometimes not possible or not desired by the landlord. This inequality is reflected in the difference between the proportion of owner-occupied and rented dwellings in which conversion measures have been carried out.^46,47^ In addition, tenants have, on average, fewer financial resources for carrying out such costly adaptations.^30^ Owners, on the other hand, are more likely to live in dwellings that have been adapted to their personal needs over time. This, and the knowledge that a person-environment mismatch is usually only considered when it occurs, leads to the expectation that the value of predictors that satisfy basic needs will be higher for tenants than for owners.

*V2 & V3: The strength of preference for a new, smaller home is greater when the conditions to fulfill basic needs (senior-friendly bathroom, elevator) are present (especially for tenants).*

Similarly, we expect both groups to be influenced by higher-order needs; however, due to natural prioritization, owners may place slightly more emphasis on these needs (eg, residential area, distance to kin). As the radius of action decreases, the need for infrastructure within walking distance may increase. It is also possible that proximity to relatives plays a greater role as the need for daily support increases. This applies to both tenants and owners, although the proportion of owners is higher in rural areas or on the outskirts of cities, which may be structurally weak.^48^ This could mean that owners attach more importance to the location of the new home.

*V4 & V5: The strength of preference for a new, smaller home is greater when the conditions to fulfill higher-order needs (preferred residential area, distance to kin) are present (especially for owner-occupiers).*

## Data and Methods

### Participants and Study Design

The study uses a convenience sample of 318 people aged between 50 and 99 from Germany. While geographic region was not an explicit inclusion criterion, the majority of participants were recruited from the northwest of Germany. This regional concentration reflects the location of the research center (Vechta in Lower Saxony) and the practical constraints of convenience sampling. Ethical approval was obtained from an ethics committee, and written informed consent was obtained from participants. Since 2018, data have been collected continuously through face-to-face interviews. We included those 264 people in our analysis sample who (a) answered at least 9 out of 10 vignettes (minus 4 participants), (b) provided complete information about their current living situation and sociodemographic data (minus 43 participants), and as this paper is about downsizing processes, (c) live in a dwelling of at least 50 m^2^ (minus 7 participants; Table 1).

**Table 1.** Descriptives on Respondent Level.

Variable	Mean	SD	Min	Max
Age in years	65.21	10.18	50	99
Rent of dwelling per person in €^a^	573.07	299.79	36.67	2500
Size of the dwelling in sqm/person	71.26	32.31	16.67	200
Size of household	2.0	0.82	1	6
Duration of residence in years	24.05	16.83	1	90
	N	%		
Gender				
Female	148	56.06		
Male	116	43.49		
Education				
Low	32	12.12		
Medium	161	60.98		
High	71	26.89		
Equivalent income (monthly)				
Low (<1250)	82	31.06		
Medium (1250-3000)	162	61.36		
High (>3000)	20	7.58		
In paid employment				
Yes	139	52.65		
No	125	47.35		
Housing tenure				
Tenant	66	25.0		
Owner	198	75.0		

*Note*. N = 264.

a Or perceived rental value if participant owns property.

**Figure 2.**
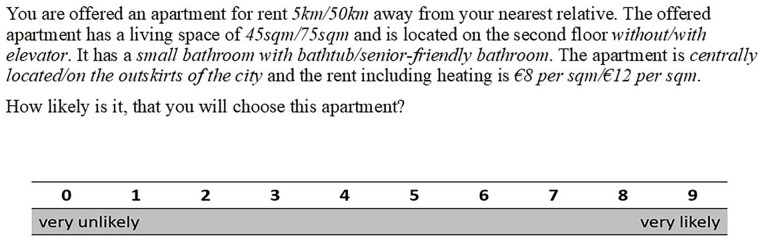
Vignette.

Participants were shown different housing descriptions (vignettes) and asked to rate them on a scale of 0 to 9. The vignettes are composed of 7 different dimensions, each with 2 values (see Figure 2), resulting in a vignette universe of 128 unique vignettes. From these 128 vignettes, 100 were randomly selected and divided into 10 sets of 10 vignettes. Each participant was shown one such set and asked to rate those 10 housing vignettes. The dimensions used in this study were derived from prior research on reasons for relocation.^2,9,49^ The number of dimensions and their respective levels were also selected in alignment with earlier studies in this field.^50-52^

Because this paper focuses on the processes involved in downsizing, we included only those vignette evaluations that described a potential move into a smaller apartment, relative to the respondent’s current living situation. We define downsizing as a reduction of at least 15 to 20 m^2^ or a decrease of around 20% in living space. These thresholds are based on empirical findings regarding downsizing patterns in later life.^53,54^ Given that even small absolute reductions can have a greater impact in already small dwellings, we operationalized downsizing as a move from at least 50 m^2^ into 45 m^2^, or—among participants living in larger homes—from at least 90 m^2^ into 75 m^2^. Based on this definition, our final analytical sample comprises 2343 vignette evaluations. Specifically, 60 participants living in homes smaller than 90 m^2^ contributed 303 ratings, while 204 participants living in homes of at least 90 m^2^ contributed 2040 ratings.

### Measurements

#### Respondent Level

We measure *employment status* using a variable (“yes,” “no,” or “in retirement”), which is collapsed into a dichotomous variable indicating “in paid employment” or “not in paid employment.” *Educational level* was assessed via 3 categories: (1) no school qualifications or low educational level (primary education), (2) intermediate educational level (lower and upper secondary education), and (3) high educational level (bachelor, master, and PhD). We use the *household equivalent net income (income* divided by the number of people living in the household). *Duration of residence* was reported in years. The *size of household refers to* the number of persons living in the apartment, including oneself. The *size of* (the current) *dwelling per person* is reported in sqm. Tenants were asked for their current *rent in euros*. Owners, in contrast, were asked to estimate the monthly rent they would expect to pay if they were renting their property. As this estimate is inherently subjective and likely influenced by perceptions of location, quality, and market knowledge, we interpret it as the *perceived rental value*.

#### Vignette Level

**Figure 3.**
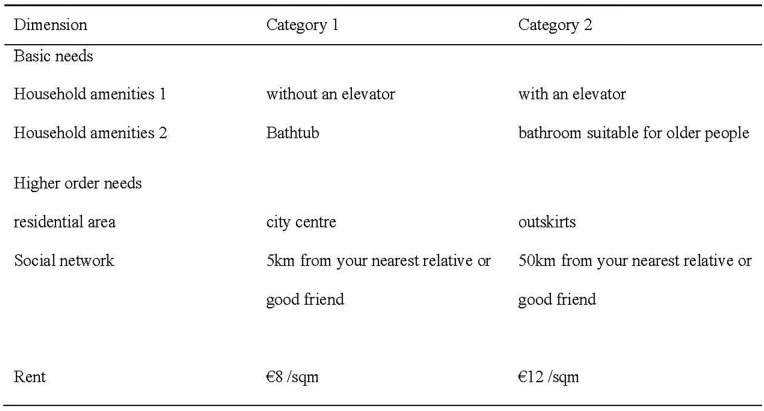
Dimensions and Categories of Vignettes.

The *preference for a new hypothetical apartment* was measured with the question “How likely is it that you will choose this apartment?” answered on a scale from 0 (“very unlikely”) to 9 (“very likely”). The dimensions dealt with in this paper are household amenities 1 and 2, residential area, social network, and rent (see Figure 3). We do not include the size of the new apartment as a dimension in our analysis, as we systematically excluded observations based on participants’ individual combinations of the size of their current home and the size of the apartment described in the vignette. One vignette dimension was experimentally varied but not included in the analysis, as it was not central to the theoretical framework and did not yield significant variation between the 2 groups in pretests. Descriptive checks confirmed that this dimension was orthogonal to other attributes and evenly distributed across vignettes. We therefore assumed that its exclusion does not introduce systematic bias in the estimates. See also Supplemental Table S4 in the supplement for more information on the number of presentations of each category.

### Analytic Strategy

**Figure 4.**
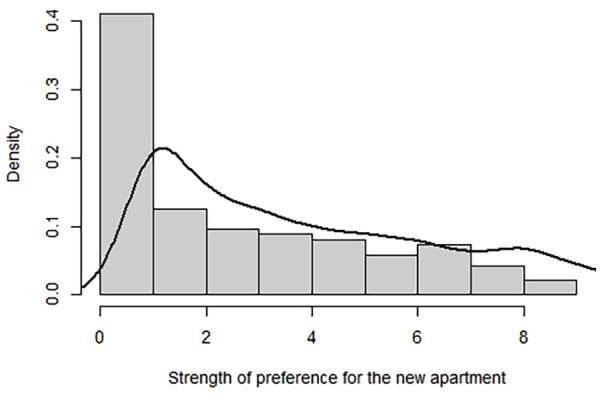
Preference for the new apartment.

For the analyses, we used R version 2023.12.1. We analyze mean differences using the *t*-test and Welch’s test. Since our dependent variable shows a high proportion of zeros (which is common in vignette studies^55^; see Figure 4), a linear regression wouldn’t fit the data distribution. The zero-inflated model acknowledges that the zero observations might have 2 different origins. One is that a participant doesn’t consider moving at all (structural origin), and the other is that a participant dislikes the specific apartment so much that they assign it zero points (sampling origin).^56^ We estimated a zero-inflated negative binomial regression model. Because participants contributed varying numbers of vignette ratings (ranging from 4 to 10), and this variation was systematically associated with housing characteristics (notably living space), we corrected for clustering at the respondent level by applying cluster-robust standard errors (by id). This approach addresses potential bias arising from non-independent observations and overrepresentation of participants with larger homes. We refrained from excluding participants with fewer ratings to avoid systematic bias against subgroups with limited data. In addition, we conducted a sensitivity check by including the number of completed vignette ratings as a covariate in the count part of the zero-inflated model. The coefficient for this covariate was not statistically significant, suggesting that the number of ratings per participant did not bias the main results. Accordingly, it was not retained in the final model. In addition to reporting effect sizes and confidence intervals based on cluster-robust standard errors (see Supplemental Table S5 in the supplement), we calculated average marginal effects (AMEs) to aid interpretation of the model results. AMEs represent the average change in the expected outcome associated with a one-unit change in a predictor, holding all other variables constant.

We compared 4 models to evaluate the best-fitting specification for our purpose: a standard Poisson model, a zero-inflated Poisson (ZIP) model, a hurdle model, and a zero-inflated negative binomial (ZINB) model. Model fit was assessed using Akaike’s Information Criterion (AIC) and Vuong’s non-nested hypothesis tests. The ZINB model yielded the lowest AIC value (AIC = 9974.52), indicating superior model fit compared to the ZIP (AIC = 10 054.45), hurdle (AIC = 10 118.22), and standard Poisson model (AIC = 10 961.41). Vuong tests confirmed the superiority of the ZINB model over all other alternatives. Specifically, the ZINB model fit significantly better than the ZIP model (*z* = –4.08, *P* < .001), the hurdle model (*z* = –6.67, *P* < .001), and the standard Poisson model (*z* = –13.00, *P* < .001). These results indicate that the ZINB model provides the best fit to the data, accommodating both overdispersion and excess zeros in the vignette ratings.

We conducted post-hoc power analyses separately for the count and zero-inflation components of the ZINB model using a bootstrap-based likelihood ratio test approach. In 500 resampled datasets, each model (count or zero) was compared against a nested intercept-only version. Statistical power was defined as the proportion of resamples in which the whole model significantly outperformed the null model (*P* < .05). The estimated power was 1.00 for the count component and 0.93 for the zero-inflation component, indicating a high probability of detecting meaningful effects.

In earlier stages of this study, downsizing was operationalized strictly as a move into a 45 m^2^ apartment, and only vignettes reflecting such a scenario were included in the analysis. These results are reported in the appendix as part of a sensitivity check. Additionally, we conducted an extended analysis that included all available vignettes—those representing moves to larger apartments as well—to examine the robustness of our findings. This extended analysis is likewise presented in the supplement (Supplemental Table S7).

We used the STROBE cross-sectional checklist when writing our report.^57^ We used the Total Error Framework to deal with potential biases.^58^ For further information, please refer to the Supplemental Material.

## Results

### Differences by Ownership

Table 2 compares tenants and owners across various variables. Compared to owners, tenants are on average older (67.8 vs 64.4 years, *P* < *.05*), have less space per person (56.8 vs 76.1 m^2^, *P* < .001), live in smaller households (1.6 vs 2.1 persons/household), and have lived in the dwelling for a shorter period (14.7 vs 27.2 years, *P* < .001). Gender, employment, and education distributions show no significant differences. Income levels reveal a larger but not statistically significant difference in the share of high-income individuals among owners (8.6% vs 0.5%, *P* = n.s.). There is no significant difference between tenants and owners in their preference for the new apartment (3.0 vs 2.8, *P* = n.s.).

**Table 2.** Mean Differences by Ownership.

	Tenant	Owner	Sign. Welch-test^a^	Sign. T-test^a^
Variable	Mean	Mean		
Age in years	67.8	64.4	*	
Size of the dwelling in m^2^/person	56.8	76.1	***	
Size of household	1.6	2.1		***
Duration of residence in years	14.7	27.2		***
Preference for a new apartment	3.0	2.8		n.s
	%	%	Sign. Pearson’s Chi-squared test	
Female	56.1	56.1	n.s	
Education				
Medium	57.6	62.1	n.s	
High	25.8	27.3		
Income				
Medium (1250-3000)	63.6	60.6	n.s	
High (>3000)	0.5	8.6		
Employed	43.9	55.5	n.s	

* *P* < .05. **P < .01. ***P < .001.

a t-test/welch-test: for variance homogeneity t-test, for variance heterogeneity welch-test.

### Prediction of Preferences

Table 3 examines the preference for a hypothetical apartment using a zero-inflated model, with separate analyses for all respondents (Model 1), tenants (Model 2), and owners (Model 3). The models include a binomial component (A) predicting the likelihood of general opting for a new apartment (0 or more than 0) and a linear component (B) estimating the strength of preference (from 0 to 9). In the A-models (A_A,_ A_T,_ A_O_), we describe which factors are contributing to participants’ general likelihood of opting for an apartment. This corresponds to the structural origin of zeros, where a participant does not consider relocation at all and therefore consistently assigns a rating of zero. Statistically speaking, we report the predictors of the likelihood of participants not always answering with zero. In the B-models (B_A_, B_T_, B_O_), we model the linear associations between the predictors and the strength of preference (0 -9), reflecting the sampling origin of zeros, which indicates that a particular apartment is intensely disliked and therefore receives zero points. Results in relation to the hypotheses are presented in Supplemental Table S6 in the Supplemental Material.

**Table 3.** Zero-Inflated Model, With Preference for a New Apartment as the Dependent Variable.

	Model 1: All						Model 2: Tenants						Model 3: Owner					
	A_A_				B_A_		A_T_			B_T_			A_O_				B_O_	
Variable	γ	AME	*p* ^a^	β	AME	*p*	γ	AME	*p*	β	AME	*p*	γ	AME	*p*	β	AME	*p*
Respondent level (R1-R5)																		
Age	−0.0	−0.00	.841	.00	0.01	.967	−0.10	−0.01	.05	.01	0.02	.617	−0.00	−0.00	.891	−.00	−0.01	.609
Gender (male = 1)	−0.02	−0.03	.937	−.04	−0.13	.736	0.29	0.02	.815	−.05	−0.16	.737	0.07	0.01	.820	−.02	−0.08	.770
Education																		
Medium	0.06	0.01	.903	.10	0.38	.293	−4.67	−0.31	<.001	−.08	−0.28	.633	0.07	0.01	.910	.07	0.25	.561
High	−0.34	−0.05	.434	.12	0.45	.288	−5.85	−0.26	<.001	−.07	−0.26	.719	−0.31	−0.04	.589	.10	0.38	.425
Size of dwelling/person	−0.0	−0.00	.885	.00	0.01	.736	0.05	0.0	.016	−.00	−0.00	.906	−0.00	−0.00	.949	.00	0.01	.721
Size of household	−0.21	−0.03	.554	.00	0.01	.956	−2.69	−0.22	.120	−.19	−0.67	.174	−0.13	−0.02	.727	.02	0.06	.830
Duration of residence	0.00	−0.00	.654	−.00	−0.01	.432	−0.16	−0.01	.004	−.01	−0.03	.145	0.01	0.00	.643	−.00	−0.00	.766
Income																		
Medium	0.31	0.04	.331	.04	0.14	.692	2.25	0.13	.006	.13	0.47	.387	0.40	0.06	.276	.03	0.12	.761
High	0.74	0.13	.256	.09	0.32	.627	−3.12	−0.13	<.001	.21	0.83	.408	0.85	0.15	.244	.06	0.23	.755
Rent or prv^b^/person	−0.01	0.00	.493	−.00	−0.00	.798												
Rent/person							0.00	0.0	.691	−.00	−0.00	.956						
Prv^b^/person													−0.00	−0.00	.469	−.00	−0.00	.806
Employed (yes = 1)	−0.64	−0.09	.054	−.04	−0.14	.588	0.55	0.04	.610	.17	0.56	.381	0.68	−0.09	.063	−.06	−0.20	.510
Ownership (yes = 1)	0.09	0.01	.808	−.04	−0.14	.548												
Vignette level (V1-V5)																		
Rent				−.22	−0.79	<.001				−.36	−1.29	<0.001				−.19	−.67	<0.001
Senior-friendly bath				.23	0.86	<.001				.26	0.95	0.002				.23	.85	<0.001
Elevator				.45	1.64	<.001				.60	2.08	<0.001				.44	1.59	<0.001
Outskirts				−.10	−0.34	.004				−.09	−0.33	0.132				−.10	−.35	0.016
Distance to kin				−.37	−1.32	<.001				−.30	−1.08	<0.001				−.38	−1.35	<0.001
Intercept	−0.50			1.22			8.00			1.23			−.69			1.28		
Observations	2343						436						1907					
AIC	9974.523						1846.097						8131.808					
BIC	10 158.82						1968.427						8298.406					
Log Likelihood	−4955.261						−893.0487						−4035.904					

*Note*. A: Binomial with logit link; B: Negbin with log link.

a *P*-value of average marginalized effects.

b Perceived rental value, measured in estimated rent if person owns property.

#### Likelihood of Opting for New Apartments (A-Models)

The likelihood of always answering with zero was higher for tenants in smaller apartments and those with a long duration of residence, as well as for tenants with a high income and a high or medium level of education (Model A_T_). In other words, tenants currently residing in larger dwellings, those with a short duration of residence, and those with a medium or low income and low education were more likely to opt for the new apartments to some extent. There were no significant predictors in the model with all participants (A_A_) or with the owners (A_O_).

#### Strength of Preference for New Apartments (B-Models)

There were no significant predictors of the strength of preference for a new apartment on the respondent level in the group of tenants (B_T_) or owners (B_O_). The cost to rent the new apartment played an essential role for both groups, but the AME is more than twice as high for the group of tenants. Participants in both groups prefer new apartments with senior-friendly bathrooms and elevators, as well as those sited close to relatives. Comparing the 2 groups, living close to relatives has a higher AME in the owner group, while having an elevator has a higher AME in the tenants group. In addition, owners (B_O_) prefer to live in the city center, while tenants do not consider the residential area when making housing decisions.

In an additional analysis that included only vignettes describing 45 m^2^ apartments, owners with high incomes were somewhat more likely to opt for the new apartment. In contrast, tenants placed even greater emphasis on the rent level while not considering the residential area and the presence of a senior-friendly bathroom. Overall, tenants in this analysis showed a stronger preference for the new apartment than owners.

## Discussion

### Summary

The primary objective of this study was to describe the differences in housing choices between homeowners and tenants. In summary, tenants tend to have a more positive attitude toward moving to a new, smaller apartment if they have not lived in their current apartment for a long time, currently reside in a larger dwelling than the proposed new one, and have a medium or low income. Owners show a high hypothetical likelihood of moving if the new home is in the city center. Both groups consider the rent (of the future apartment), proximity to relatives, and a senior-friendly bathroom when making their decisions. However, rent plays a greater role for those who are already tenants, while those who are currently owners place more value on the proximity to their relatives.

Consistent with recent findings, age did not predict apartment uptake in any of the groups. Our finding that tenants are more open to relocation when they have lived in their apartment for a short time aligns with Angelini and Laferrère’s results, which indicate that renters moving to another rental property are more likely to have spent a shorter period in their previous dwelling. Like ourselves, Angelini found no predictive value of the duration of residence for owners moving to rental properties.^10^

While Angelini and Laferrère found that homeowners in the highest income quartile are less likely to relocate,^10^ our results suggest the opposite: homeowners with high incomes appear more open to relocation when considering 45 m^2^ apartments. Similarly, our finding that tenants express a greater overall preference for new 45 m^2^ apartments seems to contradict Granbom et al’s conclusion that owners are generally more inclined to relocate.^37^ This may point to the well-documented intention-behavior gap^52^ or could be a methodological artifact. Since this finding is limited to vignettes describing small (45 m^2^) apartments—typically more familiar to tenants—the design may have led to higher initial enthusiasm among tenant participants.

### Results Against the Backdrop of Environmental Proactivity

The second aim was to discuss the results against the backdrop of environmental proactivity. Lawton and Simon described it as profitable to look at the way older people use their environment and how they attempt to alter it in their interests.^59^ The environmental proactivity hypothesis posits that as personal competence grows, individuals can access a broader range of environmental resources to meet their needs,^4^ suggesting that environmental proactivity could serve as a pathway to successful aging. However, some people may be inhibited in their environmental proactivity or experience barriers to acting on it. It is well established in the literature on health inequalities that tenants are disadvantaged in certain respects compared to homeowners. Does this trend carry into the area of environmental proactivity?

Tenants in bigger apartments and those with medium or low income were more likely to consider relocation. In addition, tenants placed a higher emphasis on the rental cost of the new apartment than owners did. This combination of findings suggests that tenants may not have sufficient financial resources to maintain an ample living space and are therefore forced to move from a potentially suitable and beloved environment to another due to economic pressures. In this case, their environmental proactivity would be limited or even prevented altogether by financial considerations. Tenants who have lived in their current apartment for a long time are more likely to reject the idea of moving outright, most likely due to a strong attachment to and sense of identity with their living environment. This finding suggests that—at least for some tenants—moving is explicitly a form of environmental proactivity because it means choosing a more (financially) suitable environment, while still considering aspects not related to financial issues.

Owners are the only group that places value on a central location. Combined with our additional analysis, which was limited to 45 m^2^ apartments, and showed that tenants did not consider a senior-friendly bathroom to be relevant, this suggests that tenants give less weight to certain aspects directly related to the physical living environment. This pattern may indicate that owners are more likely than tenants to base their relocation decisions on current housing needs.

Finally, owners with lower incomes are less likely to choose to move to 45 m^2^ apartments. Owners with lower incomes may have a particularly high financial commitment to the current home and may consider it to be particularly hard-earned. This could be an obstacle to relocation, even when the environment is no longer suitable, and therefore also an obstacle to environmental proactivity.

In summary, we can say that both groups may experience inhibition in environmental proactivity. Although the tendency is for owners to seem slightly more able to ground their decisions in housing-related parameters, we cannot say that there is a clear disadvantage for tenants in exercising environmental proactivity.

### Limitations and Future Research

First, we are measuring intentions to move house in a hypothetical situation and not real-life behavior; previous research indicates a gap between intentions and actual behavior.^9,52^ A hypothetical relocation decision, by definition, can never fully reflect the complexity underlying real-world moving decisions. In our study, it serves as a methodological tool to isolate core influencing factors under controlled conditions. This reduction of complexity is an intentional element of the study design, aimed at making specific mechanisms observable without being obscured by the multitude of interacting variables in real life. We acknowledge, however, that this operationalization excludes other common forms of downsizing observed in practice, such as relocating to smaller owner-occupied properties, moving to more affordable or better-connected neighborhoods, or prioritizing the retention of ownership. As such, autonomy-related motives and non-financial considerations may be underrepresented in our analytic framework. Furthermore, by focusing exclusively on rental apartments, our vignette design intentionally narrowed the range of housing options to those most common in later-life relocations in Germany. While this improves design clarity and respondent comparability, it may limit the generalizability of our findings to older adults for whom ownership retention or alternative housing types (eg, small single-family homes) are relevant considerations.

Second, in the past, discussions have arisen about consistency issues in factorial survey design, as vignette studies can be cognitively demanding for participants. However, it has been shown that this method is still suitable for older people, as it allows complex experimental questions to be investigated in a relatively economical design.^50,51^

Third, as this study employed a convenience sample, it has some limitations. The sample size for this study was not determined through a priori power calculations; instead, it was guided by the number of eligible individuals available for recruitment during the study period, with the general aim of exceeding the sample sizes used in comparable prior studies.^50,52^ While this pragmatic approach allowed for broad inclusion of the target population, it limits the ability to determine with certainty whether the study was adequately powered to detect all relevant effects. Nevertheless, the analytic sample of 2343 observations from 264 individuals offers a solid empirical foundation. Model diagnostics and simulation-based post hoc power approximations suggest that the data structure was well-suited to the applied statistical framework. While our convenience sample from northwest Germany limits generalizability and exhibits an imbalance between owners and tenants, the use of average marginal effects (AMEs) facilitates meaningful comparisons across groups. For future studies aiming at population-level inference or more precise estimates, particularly for tenants, probability sampling, or targeted oversampling would be beneficial.

Fourth, although the zero-inflated negative binomial model used in this study is well suited to account for overdispersion and excess zeros in the outcome variable, the potential for Type II errors should be acknowledged. The model includes a substantial number of covariates in both the count and zero-inflation components, which increases the risk of overfitting and may reduce statistical power for detecting more minor effects. Additionally, we applied cluster-robust standard errors to account for repeated observations nested within individuals. While this approach improves the robustness of inference, it tends to produce more conservative standard errors, which may obscure true associations. This limitation may reduce the model’s sensitivity to within-cluster heterogeneity. Accordingly, some effects may remain undetected due to limited statistical power. A methodological limitation of this study concerns the post hoc exclusion of a subset of data that depicted upsizing scenarios. While our focus was on typical late-life downsizing processes, the removal of these vignettes and respondents introduces a degree of selection. It may limit the generalizability of our findings to other residential trajectories in later life. Furthermore, although our experimental design included 2 apartment sizes (45 and 75 m^2^), we did not explicitly model this variable in the analysis. This decision was made due to the systematic exclusion of upsizing cases, which introduced imbalance and reduced the interpretability of apartment size as an independent predictor. Instead, we treated the relocation scenarios uniformly as reflecting downsizing. While this approach allowed us to focus on a common housing transition in old age, it may have obscured potential heterogeneity in preferences related to apartment size.

Fifth, we do not have any information on the actual housing costs of the owners (eg, heating costs, etc.), the condition of tenants’ dwellings, or more information about the individuals—such as their health status—or about their current housing situation, such as accessibility and proximity to relatives. The extended data collection period may have introduced temporal variation due to changes in housing markets or societal attitudes toward relocation. As survey date information was unavailable for most cases due to anonymization, we were unable to adjust for potential time trends. This limits comparability across cohorts and should be considered when interpreting the findings. Future research should incorporate survey timing to better account for such effects.

Sixth, we did not ask specific questions about environmental proactivity or related constructs. Accordingly, our reflections on barriers to environmental proactivity are purely theoretical. They can only serve as food for thought for further research, not as evidence of an empirically demonstrable advantage or disadvantage for either group. In our study, we are constrained by the fact that we can only model a simplified version of real-life decision-making through standardized vignettes. While this enables analytical control and comparability, it limits our ability to capture the full scope of environmental proactivity as a diverse and flexible aging strategy. Specifically, our focus on downsizing might exclude other proactive housing decisions—such as relocating for neighborhood accessibility, staying in ownership despite spatial changes, or prioritizing proximity to care networks—that do not necessarily involve a reduction in floor space. These dimensions, though conceptually central to environmental proactivity, are not represented in our data and should be explored in future research using broader, more integrative methodological approaches.

## Conclusion

Until a few years ago, remaining in the home one has lived in for years was considered the only desirable path for successful aging. However, studies now refute the generally negative association between well-being and moving in old age.^1,44,60^ In cases with high environmental pressure, a home-to-home move can lead to an improvement in person-environment fit.^1,2^ Against the backdrop of environmental proactivity, a home-to-home move can be understood as a strategy for successful aging,^1^ and has already been recognized as a primary preventive strategy.^2^ The findings from this quasi-experimental study highlight the differing preferences and considerations of homeowners and tenants in old age when it comes to agreeing to a new, smaller apartment. Homeowners are more influenced by the characteristics of their current and potential new dwelling, while tenants place greater emphasis on factors related to their financial situation and rental conditions. While the present study is situated within the German housing and policy context, the challenges it addresses—such as enabling autonomy in old age, balancing relocation decisions, and promoting environmental proactivity—are highly relevant to aging societies in general. When planning housing policy or researching relocations, factors that may influence environmental proactivity should be taken into consideration.

## Supplemental Material

sj-docx-1-inq-10.1177_00469580251370562 – Supplemental material for Owning, Renting and Environmental Proactivity: The Role of Housing Tenure in Hypothetical Housing Decisions Supplemental material, sj-docx-1-inq-10.1177_00469580251370562 for Owning, Renting and Environmental Proactivity: The Role of Housing Tenure in Hypothetical Housing Decisions by Manuela Schulz, Christiane Gross and Andrea Teti in INQUIRY: The Journal of Health Care Organization, Provision, and Financing

sj-docx-2-inq-10.1177_00469580251370562 – Supplemental material for Owning, Renting and Environmental Proactivity: The Role of Housing Tenure in Hypothetical Housing Decisions Supplemental material, sj-docx-2-inq-10.1177_00469580251370562 for Owning, Renting and Environmental Proactivity: The Role of Housing Tenure in Hypothetical Housing Decisions by Manuela Schulz, Christiane Gross and Andrea Teti in INQUIRY: The Journal of Health Care Organization, Provision, and Financing

sj-docx-3-inq-10.1177_00469580251370562 – Supplemental material for Owning, Renting and Environmental Proactivity: The Role of Housing Tenure in Hypothetical Housing Decisions Supplemental material, sj-docx-3-inq-10.1177_00469580251370562 for Owning, Renting and Environmental Proactivity: The Role of Housing Tenure in Hypothetical Housing Decisions by Manuela Schulz, Christiane Gross and Andrea Teti in INQUIRY: The Journal of Health Care Organization, Provision, and Financing

sj-docx-4-inq-10.1177_00469580251370562 – Supplemental material for Owning, Renting and Environmental Proactivity: The Role of Housing Tenure in Hypothetical Housing Decisions Supplemental material, sj-docx-4-inq-10.1177_00469580251370562 for Owning, Renting and Environmental Proactivity: The Role of Housing Tenure in Hypothetical Housing Decisions by Manuela Schulz, Christiane Gross and Andrea Teti in INQUIRY: The Journal of Health Care Organization, Provision, and Financing

sj-docx-5-inq-10.1177_00469580251370562 – Supplemental material for Owning, Renting and Environmental Proactivity: The Role of Housing Tenure in Hypothetical Housing Decisions Supplemental material, sj-docx-5-inq-10.1177_00469580251370562 for Owning, Renting and Environmental Proactivity: The Role of Housing Tenure in Hypothetical Housing Decisions by Manuela Schulz, Christiane Gross and Andrea Teti in INQUIRY: The Journal of Health Care Organization, Provision, and Financing

sj-docx-6-inq-10.1177_00469580251370562 – Supplemental material for Owning, Renting and Environmental Proactivity: The Role of Housing Tenure in Hypothetical Housing Decisions Supplemental material, sj-docx-6-inq-10.1177_00469580251370562 for Owning, Renting and Environmental Proactivity: The Role of Housing Tenure in Hypothetical Housing Decisions by Manuela Schulz, Christiane Gross and Andrea Teti in INQUIRY: The Journal of Health Care Organization, Provision, and Financing
