# Supplementary material for: Owning, Renting and Environmental Proactivity: The Role of Housing Tenure in Hypothetical Housing Decisions
Source: Inquiry. 2025 Sep 15;62:00469580251370562. doi: 10.1177/00469580251370562 (PMC12437166; doi:10.1177/00469580251370562)
Supplement: sj-docx-1-inq-10.1177_00469580251370562 – Supplemental material for Owning, Renting and Environmental Proactivity: The Role of Housing Tenure in Hypothetical Housing Decisions [file sj-docx-1-inq-10.1177_00469580251370562.docx]

**Potential Biases**

To address potential biases, we use the total survey error framework (TSE) ^1^ . The TSE is a comprehensive approach to evaluating and minimizing errors in survey research. It categorizes error sources into two main branches: Measurement and Representation. Measurement errors include issues related to construct validity, survey questionnaire design, and processing errors. Representation errors encompass problems with coverage, sample design, and nonresponse. By systematically addressing these error sources, the TSE framework aims to optimize the balance between error reduction and practical constraints like budget and timelines, ensuring the collection of accurate and reliable data.

Validity and measurement errors: In this study, participants are asked to make a hypothetical decision. When interpreting the results, it is therefore important to bear in mind that there may be a certain discrepancy between what is said and what is actually done. In order to create a professional interview situation, the interviewers were trained in detail on the topics: ‘Conducting interviews and communication’, ‘Sensitivity to stressful situations’ ‘Closeness and distance in the research context’, and ‘Reliable data and research quality’.

Processing errors: The data were collected in a face-to-face interview. In this interview the answers were recorded in pen and ink. In order to avoid errors due to handwriting, the questions were developed in a tick box format.

Analytical errors: The data were cleaned and analyzed using R. The script was discussed regularly between the authors and is available for review.

Coverage errors: This study uses convenience data. It is therefore not possible to speak of a representative representation of the age group. However, as the study also deals with a question that is more relevant to healthy older people, the sample can be considered representative of the relevant group.

Sampling errors: The number of participants in our study exceeds that of other studies using this method. It can therefore be assumed that there is no particular sampling error.

Non-response errors: We found that the variable with the highest non-response rate was the income variable. Unfortunately, this is a common phenomenon in the social sciences.

References

1. Groves RM, Lyberg L. Total Survey Error: Past, Present, and Future. *Public Opinion Quarterly*. 2010;74(5):849-879. doi:10.1093/poq/nfq065
