# Supplementary material for: Owning, Renting and Environmental Proactivity: The Role of Housing Tenure in Hypothetical Housing Decisions
Source: Inquiry. 2025 Sep 15;62:00469580251370562. doi: 10.1177/00469580251370562 (PMC12437166; doi:10.1177/00469580251370562)
Supplement: sj-docx-3-inq-10.1177_00469580251370562 – Supplemental material for Owning, Renting and Environmental Proactivity: The Role of Housing Tenure in Hypothetical Housing Decisions [file sj-docx-3-inq-10.1177_00469580251370562.docx]

|  | Number of presentations | |
| --- | --- | --- |
|  | 2343 observations^1^ | 2639 observations^2^ |
| Household amenities 1 |  |  |
| without an elevator | 1196 | 1347 |
| with an elevator | 1147 | 1292 |
| Household amenities 2 |  |  |
| bathtub | 1291 | 1446 |
| bathroom suitable for older people | 1052 | 1193 |
| Residential area |  |  |
| city centre | 1144 | 1288 |
| outskirts | 1199 | 1351 |
| Social network |  |  |
| 5km from your nearest relative or good friend | 1238 | 1391 |
| 50km from your nearest relative or good friend | 1105 | 1248 |
| Rent |  |  |
| €8 /sqm | 1160 | 1318 |
| €12 /sqm | 1183 | 1321 |
| ^1^ only vignettes representing a downsizing move for the individual  ^2^ all vignettes for every participant | | |

Table S4: Number of presentations of vignette categories
