# Supplementary material for: Owning, Renting and Environmental Proactivity: The Role of Housing Tenure in Hypothetical Housing Decisions
Source: Inquiry. 2025 Sep 15;62:00469580251370562. doi: 10.1177/00469580251370562 (PMC12437166; doi:10.1177/00469580251370562)
Supplement: sj-docx-4-inq-10.1177_00469580251370562 – Supplemental material for Owning, Renting and Environmental Proactivity: The Role of Housing Tenure in Hypothetical Housing Decisions [file sj-docx-4-inq-10.1177_00469580251370562.docx]

Table S5: Zero inflated model with effect sizes and CI

|  | | Model 1: All | | | | | | | | | | | | | Model 2: Tenants | | | | | | | | | | | | | | Model 3: Owner | | | | | | | | | | | | | |
| --- | --- | --- | --- | --- | --- | --- | --- | --- | --- | --- | --- | --- | --- | --- | --- | --- | --- | --- | --- | --- | --- | --- | --- | --- | --- | --- | --- | --- | --- | --- | --- | --- | --- | --- | --- | --- | --- | --- | --- | --- | --- | --- |
|  | | A_A_ | | | | | | | B_A_ | | | | | | A_T_ | | | | | | | | | B_T_ | | | | | A_O_ | | | | | | | | | B_O_ | | | | |
|  | | γ | | OR | | 95%-CI | | β | | | RR | | 95%-CI | | | γ | | | OR | | 95%-CI | | β | | RR | | | 95%-CI | | γ | | | OR | | 95%-CI | | β | | | RR | | 95%-CI |
| Respondent level (R1-R5) | |  | |  | |  | |  | | |  | |  | | |  | | |  | |  | |  | |  | | |  | |  | | |  | |  | |  | | |  | |  |
| Age | | -0.0 | | 0.997 | | [0.966, 1.029] | | 0.00 | | | 1.000 | | [0.993, 1.008] | | | -0.10 | | | 0.904 | | [0.803, 1.019] | | 0.01 | | 1.005 | | | [0.986, 1.024] | | -0.00 | | | 0.997 | | [0.959, 1.037] | | -0.00 | | | 0.998 | | [0.990, 1.006] |
| Gender (male = 1) | | -0.02 | | 0.981 | | [0.863, 1.593] | | -0.04 | | | 0.964 | | [0.865, 1.075] | | | 0.29 | | | 1.336 | | [0.105, 17.056] | | -0.05 | | 0.956 | | | [0.733, 1.246] | | 0.07 | | | 1.068 | | [0.596, 1.915] | | -0.02 | | | 0.979 | | [0.865, 1.128] |
| Education | |  | |  | |  | |  | | |  | |  | | |  | | |  | |  | |  | |  | | |  | |  | | |  | |  | |  | | |  | |  |
| medium | | 0.06 | | 1.060 | | [0.407, 2.759] | | 0.10 | | | 1.107 | | [0.911, 1.345] | | | -4.67 | | | 0.009 | | [0.0, 1.781] | | -0.08 | | 0.926 | | | [0.675, 1.269] | | 0.07 | | | 1.068 | | [0.331, 3.447] | | 0.07 | | | 1.072 | | [0.842, 1.366] |
| high | | -0.34 | | 0.687 | | [0.268, 1.758] | | 0.12 | | | 1.127 | | [0.905, 1.403] | | | -5.85 | | | 0.003 | | [0.0, 0.412] | | -0.07 | | 0.929 | | | [0.620, 2.393] | | -0.31 | | | 0.733 | | [0.240, 2.240] | | 0.10 | | | 1.108 | | [0.860, 1.428] |
| Size of dwelling/person | | -0.0 | | 0.999 | | [0.983, 1.015] | | 0.00 | | | 1.002 | | [0.992, 1.012] | | | 0.05 | | | 1.056 | | [1.019, 1.094] | | -0.00 | | 1.000 | | | [0.992, 1.007] | | -0.00 | | | 0.999 | | [0.981, 1.018] | | 0.00 | | | 1.003 | | [0.989, 1.016] |
| Size of household | | -0.21 | | 0.831 | | [0.41, 1.613] | | 0.00 | | | 1.004 | | [0.872, 1.156] | | | -2.69 | | | 0.068 | | [0.001, 8.373] | | -0.19 | | 0.828 | | | [0.633, 1.082] | | -0.13 | | | 0.874 | | [0.418, 1.828] | | 0.02 | | | 1.017 | | [0.867, 1.194] |
| Duration of residence | | 0.00 | | 1.004 | | [0.988, 1.02] | | -0.00 | | | 0.998 | | [0.994, 1.003] | | | -0.16 | | | 0.855 | | [0.736, 0.994] | | -0.01 | | 0.993 | | | [0.983, 1.002] | | 0.01 | | | 1.005 | | [0.985, 1.025] | | -0.00 | | | 0.999 | | [0.994, 1.005] |
| Income | |  | |  | |  | |  | | |  | |  | | |  | | |  | |  | |  | |  | | |  | |  | | |  | |  | |  | | |  | |  |
| medium | | 0.31 | | 1.363 | | [0.733, 2.534] | | 0.04 | | | 1.040 | | [0.86, 1.257] | | | 2.25 | | | 9.521 | | [1.268, 71.468] | | 0.13 | | 1.143 | | | [0.843, 1.549] | | 0.40 | | | 1.491 | | [0.716, 3.104] | | 0.03 | | | 1.034 | | [0.834, 1.282] |
| high | | 0.74 | | 2.093 | | [0.65, 6.741] | | 0.09 | | | 1.088 | | [0.788, 1.502] | | | -3.12 | | | 0.041 | | [0.0, 14.388] | | 0.21 | | 1.237 | | | [0.784, 1.952] | | 0.85 | | | 2.349 | | [0.592, 9.317] | | 0.06 | | | 1.063 | | [0.734, 1.540] |
| Rent or v  prv^2^/person | | -0.01 | | 0.999 | | [0.998, 1.001] | | -0.00 | | | 1.000 | | [0.998, 1.002] | | |  | | |  | |  | |  | |  | | |  | |  | | |  | |  | |  | | |  | |  |
| Rent/person | |  | |  | |  | |  | | |  | |  | | | 0.00 | | | 1.001 | | [0.994, 1.009] | | -0.00 | | 1.000 | | | [0.999, 1.001] | |  | | |  | |  | |  | | |  | |  |
| Prv^2^/person | |  | |  | |  | |  | | |  | |  | | |  | | |  | |  | |  | |  | | |  | | -0.00 | | | 0.999 | | [0.998, 1.001] | | -0.00 | | | 1.000 | | [0.997, 1.002] |
| Employed (yes = 1) | | -0.64 | | 0.531 | | [0.253, 1.114] | | -0.04 | | | 0.964 | | [0.842, 1.103] | | | 0.55 | | | 1.733 | | [0.202, 14.888] | | 0.17 | | 1.182 | | | [0.815, 1.713] | | 0.68 | | | 0.507 | | [0.208, 1.236] | | -0.06 | | | 0.947 | | [0.803, 1.117] |
| Ownership (yes = 1) | | 0.09 | | 1.091 | | [0.53, 2.245] | | -0.04 | | | 0.963 | | [0.853, 1.087] | | |  | | |  | |  | |  | |  | | |  | |  | | |  | |  | |  | | |  | |  |
|  | | | | | | | | | | | | | | | | | | | | | | | | | | | | | | | | | | | | | | | | | | |
| Vignette level  (V1-V5) | | |  | |  | |  | | |  | |  | |  | | |  | | |  | |  | |  | |  |  | |  | | |  | |  | |  | | |  | |  | |
| Rent | | |  | |  | |  | | | -0.22 | | 0.805 | | [0.715, 0.865] | | |  | | |  | |  | | -0.36 | | 0.696 | [0.586, 0.827] | |  | | |  | |  | | -0.19 | | | 0.831 | | [0.770, 0.896] | |
| Seniorfriendly bath | | |  | |  | |  | | | 0.23 | | 1.265 | | [1.156, 1.383] | | |  | | |  | |  | | 0.26 | | 1.299 | [1.108, 1.523] | |  | | |  | |  | | 0.23 | | | 1.263 | | [1.135, 1.405] | |
| Elevator | | |  | |  | |  | | | 0.45 | | 1.574 | | [1.344, 1.843] | | |  | | |  | |  | | 0.60 | | 1.822 | [1.453, 2.286] | |  | | |  | |  | | 0.44 | | | 1.553 | | [1.301, 1.854] | |
| Outskirts | | |  | |  | |  | | | -0.10 | | 0.908 | | [0.846, 1.974] | | |  | | |  | |  | | -0.09 | | 0.910 | [0.805, 1.029] | |  | | |  | |  | | -0.10 | | | 0.908 | | [0.834, 0.989] | |
| Distance to kin | | |  | |  | |  | | | -0.37 | | 0.691 | | [0.625, 0.764] | | |  | | |  | |  | | -0.30 | | 0.738 | [0.613, 0.888] | |  | | |  | |  | | -0.38 | | | 0.683 | | [0.606, 0.769] | |
| Intercept | | | -0.50 | | 0.606 | | [0.18, 20.285] | | | 1.23 | | 3.380 | | [1.373, 8.318] | | | 8.00 | | | 2988.501 | | [0.004, 2197090776.183] | | 1.23 | | 3.418 | [0.535, 21.841] | | -0.7 | | | 0.501 | | [0.006, 40.830] | | 1.28 | | | 3.604 | | [1.369, 9.486] | |
| Observations | 2343 | | | | | | | | | | | | | | | | | 436 | | | | | | | | | | | | | 1907 | | | | | | | | | | | |
| AIC | 9974.523 | | | | | | | | | | | | | | | | | 1846.097 | | | | | | | | | | | | | 8131.808 | | | | | | | | | | | |
| BIC | 10158.82 | | | | | | | | | | | | | | | | | 1968.427 | | | | | | | | | | | | | 8298.406 | | | | | | | | | | | |
| Log Likelihood | -4955.261 | | | | | | | | | | | | | | | | | -893.0487 | | | | | | | | | | | | | -4035.904 | | | | | | | | | | | |
| ^2^perceived rental value, measured in estimated rent if person owns property  A: Binomial with logit link, B: Negbin with log link | | | | | | | | | | | | | | | | | | | | | | | | | | | | | | | | | | | | | | | | | | |
