# Supplementary material for: Owning, Renting and Environmental Proactivity: The Role of Housing Tenure in Hypothetical Housing Decisions
Source: Inquiry. 2025 Sep 15;62:00469580251370562. doi: 10.1177/00469580251370562 (PMC12437166; doi:10.1177/00469580251370562)
Supplement: sj-docx-5-inq-10.1177_00469580251370562 – Supplemental material for Owning, Renting and Environmental Proactivity: The Role of Housing Tenure in Hypothetical Housing Decisions [file sj-docx-5-inq-10.1177_00469580251370562.docx]

Table S6: Results in relation to hypotheses

|  | Results | Group differences | Confirmation of hypothesis |
| --- | --- | --- | --- |
| Hypotheses on respondent level |  |  |  |
| *R1: The* *strength of preference for a new, smaller home is greater when the current dwelling is larger (especially for those who are currently owner-occupiers).* | AME(A_T_)=0.00 | only for tenants | no |
| *R2: The strength of preference for a new, smaller home is greater when the household size is low (especially for those who are currently owner-occupiers).* | - | - | no |
| *R3: The strength of preference for a new, smaller home is greater when the duration of residence in the current home is shorter.* | AME(A_T_)=-0.01 | only for tenants | partly |
| *R4: Tenants’ strength of preference for a new, smaller home is greater when the income is low.* | AME(A_T_)=0.13 (medium income);  AME(A_T_)=-0.13 (high income) | - | no |
| *R5: Tenants’ strength of preference for a new, smaller home is greater when the rent in the current dwelling is high.* | - | - | no |
| Hypotheses on vignette level |  |  |  |
| *V1: The strength of preference for a new, smaller home is greater when the new rent is lower (especially for tenants).* | AME(B_A_)=-0.79 | twice as high for tenants | yes |
| *V2 & V3: The strength of preference for a new, smaller home is greater when the conditions to fulfill basic needs are present (especially for tenants).* |  |  |  |
| *senior-friendly bathroom* | AME(B_A_)=0.23 | similar in both groups | partly |
| *elevator* | AME(B_A_)=0.45 | higher for tenants | yes |
| *V4 & V5: The strength of preference for a new, smaller home is greater when the conditions to fulfill higher order needs are present (especially for owner- occupiers).* |  |  |  |
| *preferred residential area* | AME(B_O_)=-0.10 | only for owner-occupiers | yes |
| *distance to kin* | AME(B_A_)=-0.37 | higher for owner-occupiers | yes |
