# Supplementary material for: Owning, Renting and Environmental Proactivity: The Role of Housing Tenure in Hypothetical Housing Decisions
Source: Inquiry. 2025 Sep 15;62:00469580251370562. doi: 10.1177/00469580251370562 (PMC12437166; doi:10.1177/00469580251370562)
Supplement: sj-docx-6-inq-10.1177_00469580251370562 – Supplemental material for Owning, Renting and Environmental Proactivity: The Role of Housing Tenure in Hypothetical Housing Decisions [file sj-docx-6-inq-10.1177_00469580251370562.docx]

Table S7: Additional Analysis

|  | Model 1: All observations, including upsizing decisions | | | | | | Model 2: Only vignettes describing small apartments | | | | | | | |
| --- | --- | --- | --- | --- | --- | --- | --- | --- | --- | --- | --- | --- | --- | --- |
|  | All | | Tenants | | Owner | | All | | Tenants | | | Owner | | |
|  | A | B | A | B | A | B | A | B | A | B | | A | | B |
|  | AME | AME | AME | AME | AME | AME | AME | AME | AME | AME | | AME | | AME |
| Respondent level (R1-R5) |  |  |  |  |  |  |  |  |  |  | |  | |  |
| Age | -0.00 | 0.00 | -0.00 | 0.02 | -0.00 | -0.00 | -0.00 | 0.00 | -0.00 | 0.01 | | -0.00 | | 0.01 |
| Gender (male = 1) | 0.00 | -0.11 | -0.01 | -0.15 | 0.01 | -0.07 | 0.01 | -0.25 | 0.05 | -0.46 | | 0.03 | | -0.10 |
| Education |  |  |  |  |  |  |  |  |  |  | |  | |  |
| medium | 0.03 | 0.45 | -0.02 | 0.04 | 0.00 | 0.34 | -0.03 | 0.27 | -0.32*** | -0.39 | | -0.00 | | 0.34 |
| high | -0.04 | 0.56 | -0.16 | -0.05 | -0.04 | 0.51 | -0.09 | 0.45 | -0.29** | -0.15 | | -0.07 | | 0.42 |
| Size of dwelling/person | -0.00 | 0.00 | -0.00 | -0.01 | -0.00 | 0.01 | 0.00 | 0.01* | 0.00 | 0.00 | | 0.00 | | 0.02 |
| Size of household | -0.03 | -0.03 | -0.34 | -0.45 | -0.02 | 0.04 | -0.08 | -0.08 | -0.22 | -0.56 | | -0.02 | | 0.10 |
| Duration of residence | 0.00 | -0.01 | -0.00 | -0.02 | 0.00 | -0.00 | 0.00 | 0.00 | -0.01 | 0.18 | | 0.00 | | 0.00 |
| Income |  |  |  |  |  |  |  |  |  |  | |  | |  |
| medium | 0.03 | 0.03 | -0.07 | -0.14 | 0.06 | 0.10 | 0.12* | 0.59*** | 0.12*** | 0.26 | | 0.13 | | 0.62 |
| high | 0.11 | 0.21 | -0.15 | 0.39 | 0.16 | 0.19 | 0.34* | 1.13 | 0.14 | 0.40 | | 0.35* | | 0.99 |
| Rent or prv^2^/person | -0.00 | -0.00 |  |  |  |  | -0.00* | -0.00 |  |  | |  | |  |
| Rent/person |  |  | 0.00 | 0.00 |  |  |  |  | 0.00 | -0.00 | |  | |  |
| Prv^2^/person |  |  |  |  | -0.00 | -0.00 |  |  |  |  | | -0.00 | | -0.00 |
| Employed (yes = 1) | -0.08 | -0.13 | 0.04 | 0.26 | -0.10 | -0.16 | -0.06 | -0.27 | 0.07 | 0.56 | | -0.09 | | -0.41 |
| Ownership (yes = 1) | 0.02 | -0.15 |  |  |  |  | -0.01 | -0.67* |  |  | |  | |  |
|  | | | | | | | | | | | | | | |
| Vignette level  (V1-V5) |  |  |  |  |  |  |  |  |  | |  | |  |  |
| Rent |  | -0.85*** |  | -1.39*** |  | -0.68*** |  | -0.64*** |  | | -1.27*** | |  | -0.47* |
| Seniorfriendly bath |  | 0.87*** |  | 0.94*** |  | 0.88*** |  | 0.95*** |  | | 0.63 | |  | 1.09*** |
| Elevator |  | 1.64*** |  | -1.72*** |  | 1.61*** |  | 1.49*** |  | | 1.77*** | |  | 1.42*** |
| Outskirts |  | -0.34* |  | -0.35 |  | -0.35* |  | -0.46** |  | | -0.29 | |  | -0.53** |
| Distance to kin |  | -1.35*** |  | -1.19*** |  | -1.37*** |  | -1.31*** |  | | -0.93* | |  | -1.39*** |
| Observations | 2639 | | 659 | | 1980 | | 1351 | | 325 | | | | 1026 | |
| AIC | 11328.86 | | 2818.507 | | 8436.059 | | 5629.521 | | 1386.559 | | | | 4243.494 | |
| BIC | 11511.09 | | 2953.229 | | 8603.785 | | 5796.196 | | 1500.073 | | | | 4391.497 | |
| Log Likelihood | -5633.432 | | -1379.253 | | -4188.03 | | -2782.76 | | -663.2793 | | | | -2091.747 | |
| ^1^ *p<.05 **p<.01 ***p<.001  ^2^perceived rental value, measured in estimated rent if person owns property  A: Binomial with logit link, B: Negbin with log link | | | | | | | | | | | | | | |
